# Differential Expression of Intestinal Genes in Opossums with High and Low Responses to Dietary Cholesterol

**DOI:** 10.1155/2010/415075

**Published:** 2009-11-23

**Authors:** Jeannie Chan, Rampratap S. Kushwaha, Jane F. VandeBerg, Jelica Gluhak-Heinrich, John L. VandeBerg

**Affiliations:** ^1^Southwest National Primate Research Center, Southwest Foundation for Biomedical Research, P.O. Box 760549, San Antonio, TX 78245-0549, USA; ^2^Department of Genetics, Southwest Foundation for Biomedical Research, P.O. Box 760549, San Antonio, TX 78245-0549, USA; ^3^Department of Orthodontics, The University of Texas Health Science Center at San Antonio, 7703 Floyd Curl Drive, San Antonio, TX 78229-3900, USA

## Abstract

High and low responding opossums (*Monodelphis domestica*) differ in their plasma very low density lipoprotein and low density lipoprotein (VLDL+LDL) cholesterol concentrations when they consume a high cholesterol diet, which is due in part to absorption of a higher percentage of dietary cholesterol in high responders. We compared the expression of a set of genes that influence cholesterol absorption in high and low responders fed a basal or a high cholesterol and low fat (HCLF) diet. Up-regulation of the *ABCG5*, *ABCG8*, and *IBABP* genes by the HCLF diet in high and low responders may reduce cholesterol absorption to maintain cholesterol homeostasis. Differences in expression of the phospholipase genes (*PLA2* and *PLB*) and phospholipase activity were associated with differences in cholesterol absorption when opossums were fed cholesterol-enriched diets. Higher *PLA2* and *PLB* mRNA levels and higher phospholipase activity may increase cholesterol absorption in high responders by enhancing the release of cholesterol from bile salt micelles for uptake by intestinal cells.

## 1. Introduction

Elevated level of plasma low density lipoprotein (LDL) cholesterol is a risk factor for atherosclerosis, and dietary cholesterol can increase plasma LDL cholesterol levels. Partially inbred strains of the laboratory opossum (*Monodelphis domestica*) have been developed to study diet-induced hypercholesterolemia. Their plasma very low density lipoprotein and low density lipoprotein (VLDL+LDL) cholesterol levels are similar on a basal diet, but the levels are markedly different on cholesterol-enriched diets because plasma VLDL+LDL cholesterol is highly elevated (>10-fold) in high responding opossums, whereas plasma VLDL+LDL cholesterol is only slightly elevated (<2-fold) in low responding opossums [1]. Analysis of lipoprotein cholesterol concentrations from pedigreed families led to the implication of a recessive gene that is largely responsible for diet-induced hypercholesterolemia [2], but the causative gene has not yet been identified.

We conducted a study to measure cholesterol absorption which showed no difference in fractional cholesterol absorption between high and low responders on the basal diet. When the opossums were switched to a high cholesterol and high fat (HCHF) diet, fractional cholesterol absorption decreased by 50% in low responders, but not in high responders [3]. Another study with opossums fed a high cholesterol and low fat (HCLF) diet also revealed that fractional cholesterol absorption was 2-fold higher in high responders compared with low responders [4]. Furthermore, treatment of high responders with the cholesterol absorption inhibitor ezetimibe reduced their plasma cholesterol levels while they consumed the HCLF diet [4]. Taken together, these studies demonstrate that intestinal cholesterol absorption is dysregulated in high responding opossums when they consume high cholesterol diets and thereby their plasma VLDL+LDL cholesterol becomes elevated.

Expression of lipases, sterol transporters, and cholesterol esterifying enzymes can modulate cholesterol absorption in the small intestine [5–7]. This study was undertaken to identify the genes whose expression is associated with the difference in cholesterol absorption. The mRNA levels of cholesterol transporters and various enzymes in the intestines of opossums fed the basal or the HCLF diet were determined by quantitative reverse transcription-polymerase chain reaction (qRT-PCR). Furthermore, a suppression subtractive hybridization was carried out to identify additional differentially expressed genes that are related to cholesterol absorption.

## 2. Materials and Methods

### 2.1. Animals and Diets

Three partially inbred strains of laboratory opossums were selectively bred for high and low responsiveness to dietary lipids at the Southwest Foundation for Biomedical Research (SFBR). The hyperresponsive opossums were from the ATHH strain, and the hyporesponsive opossums were from the ATHE and ATHL strains. Animals were maintained in polycarbonate cages under laboratory conditions as described previously [8]. Six high and six low responding opossums were fed the basal diet which contained 0.16% cholesterol (g/kg; dry weight basis); and six high and six low responding opossums were fed the HCLF diet which had the same fat content as the basal diet but a cholesterol content of 0.71% [1]. After 4 weeks on the basal or HCLF diet, animals were bled to determine plasma lipoprotein cholesterol concentrations. However, one high responding opossum on the basal diet and one low responding opossum on the HCLF diet died before tissue collection. 

Total plasma cholesterol and high density lipoprotein cholesterol concentrations were measured by methods as described previously [1]. VLDL+LDL cholesterol concentration was calculated as total plasma cholesterol concentration minus high density lipoprotein cholesterol concentration. On the HCLF diet, VLDL+LDL cholesterol of high responders was >300 mg/dL, whereas VLDL+LDL cholesterol of low responders was ~30 mg/dL.

The protocol of these experiments was approved by the Institutional Animal Care and Use Committee of the SFBR. The SFBR is accredited by the Association for Assessment and Accreditation of Laboratory Animal Care International, and is registered with the US Department of Agriculture.

### 2.2. Isolation of Intestinal RNA

The small intestine from the pyloric valve to the cecum was removed, divided into six segments of equal length, and snap frozen in liquid nitrogen. The third and fourth segments from the proximal end were designated as the jejunum, and total RNA was isolated from the third segment using the TRI Reagent (Molecular Research Center, Cincinnati, OH) according to the manufacturer's instructions. Poly A^+^RNA was isolated from total RNA using a MicroPoly(A)Purist Kit (Applied Biosystems, Foster City, CA).

### 2.3. Cloning of Differentially Expressed Genes

Differentially expressed genes in the jejunum of a high responding opossum and a low-responding opossum fed the HCLF diet were cloned by suppression subtractive hybridization (SSH) in combination with mirror orientation selection (MOS) [9]. The SSH method is based on suppression PCR for normalization and subtraction, and has been shown to enrich for novel differentially expressed genes [10–12]. 

cDNA synthesis and SSH were performed using a PCR-Select cDNA Subtraction Kit (Clontech, Mountain View, CA) according to the manufacturer's protocol. Briefly, 2 *μ*g of intestinal poly A^+^RNA isolated from each of the high responder and low responder was reverse-transcribed into cDNA, digested with *Rsa* I, and tester cDNAs were ligated with adaptors. In the forward subtraction, tester cDNAs were from the low responder and driver cDNAs were from the high responder, so the forward subtraction enriched for genes that were up-regulated in low responders. In the reverse subtraction, tester cDNAs were from the high responder and driver cDNAs were from the low responder, so the reverse subtraction enriched for genes that were up-regulated in high responders. Subtracted cDNAs were amplified by PCR according to the MOS protocol to increase the efficiency of cloning differentially expressed genes [9], and the PCR products were cloned into the pCR4-TOPO vector (Invitrogen, Carlsbad, CA).

Differential screening of the two subtracted cDNA libraries was carried out to eliminate background clones [9]. Briefly, cDNA inserts of bacterial colonies were amplified by PCR, and the PCR products were spotted on Hybond-XL membranes (GE Healthcare, Piscataway, NJ) in duplicates. The forward-subtracted and reverse-subtracted cDNAs were labeled with [*α*-^33^P]dCTP (3000 Ci/mmol; PerkinElmer, Shelton, CT) using a Megaprime DNA Labeling Kit (GE Healthcare). One membrane of each pair of identical cDNA dot blots was hybridized with the radiolabeled forward-subtracted probe and the other with the radiolabeled reverse-subtracted probe in ULTRAhyb (Ambion, Austin, TX) according to the manufacturer's instructions. Clones that showed differential signals upon screening with the two subtracted cDNA probes were sequenced using a BigDye Terminator v3.1 Cycle Sequencing Kit (Applied Biosystems), and the samples were analyzed on an ABI Prism 3100 DNA sequencer (Applied Biosystems). BLAST searches against the GenBank database were performed to reveal the identity of the genes.

### 2.4. Quantitative RT-PCR (qRT-PCR)

Gene expression was quantified by qRT-PCR using SYBR Green chemistry on a 7900HT Fast Real-Time PCR System (Applied Biosystems). Primers for real-time PCR (Table 1) were designed following the guidelines in the Fast SYBR Green Master Mix protocol from Applied Biosystems using the Primer3 program [13]. A dissociation curve analysis was performed to ensure that only a single product was amplified for each gene.

Total RNA isolated using the TRI Reagent was treated with DNase from the TURBO DNA-free Kit (Applied Biosystems). Single-stranded cDNA was synthesized from 1 *μ*g of DNase-treated RNA in a 20 *μ*L reaction using a High Capacity cDNA Reverse Transcription Kit with random primers (Applied Biosystems). The reverse transcription reaction was diluted 30-fold for genes expressed at low levels or 150-fold for genes expressed at moderate levels, and 3 *μ*L of the diluted reaction was added to a mixture (7 *μ*L) containing gene-specific primers and Fast SYBR Green Master Mix (Applied Biosystems) for PCR amplification according to the manufacturer's instructions. A standard curve was generated for each gene by serial dilutions (10-fold) of cDNAs pooled from high and low responders. Messenger RNA expression was determined from the standard curve, normalized to *GAPDH* mRNA, and expressed as arbitrary units. Results were presented as means ± S.D. Differences between experimental groups were determined using the Student's *t*-test, and the level of significance was set at *P* < .05.

### 2.5. In Situ Hybridization

A 1198 bp *PLB* cDNA was amplified by RT-PCR using a forward primer (5′-GGTAATGGAGCAGGATCTACG-3′) and a reverse primer (5′-TATTAGGCAAGGTGGTCACAC-3′), and ligated with the pcDNA6/V5-His A vector (Invitrogen) at the *Eco *RV site. One clone, S3, carried the *PLB* cDNA in the normal orientation, and was used to generate the sense probe. The other clone, AS8, carried the *PLB* cDNA in the reverse orientation, and was used to generate the antisense probe. After the plasmids were linearized by *Xba *I, RNA probes were synthesized using T7 RNA polymerase and [*α*-^32^P]rUTP (800 Ci/mmol; GE Healthcare). Following in vitro transcription, riboprobes were fragmented to an average size of 200–300 nucleotides by limited alkaline hydrolysis.

The dissected intestinal tissues were fixed in 4% paraformaldehyde overnight, then they were dehydrated in increasing concentrations of methanol while the samples were kept on ice throughout the procedure. Tissue samples were embedded in paraffin, and sectioned at 6–8 mm thickness. In situ hybridization was performed as described previously [14].

### 2.6. Intestinal Phospholipase Activity in High and Low Responding Opossums

The small intestines were from a previous study in which cholesterol absorption in high and low responders fed the HCHF diet was determined [3]. The small intestine was divided into three equal segments, and the middle segment was used for the isolation of microsomes. Microsomes were prepared by differential centrifugation as described previously [15]. After ultracentrifugation, the pellet was resuspended in 10 mmol/L HEPES, pH 7.4 (Sigma Aldrich, St. Louis, MO), and stored at −80°C until use. Protein concentration was measured by the Bio-Rad protein assay (Bio-Rad, Hercules, CA) using bovine serum albumin as a standard.

Phospholipase activity was determined by incubating microsomes (200 *μ*g) in 1 mL of buffer consisting of 1% sodium cholate, 50 mmol/L HEPES (pH 8), 1 mmol/L egg yolk phosphatidylcholine (Sigma-Aldrich), and 2 *μ*L of the radioactive substrate L-3-phosphatidylcholine, 1-palmitoyl-2-[1-^14^C]linoleoyl (GE Healthcare) at 37°C for 1 hour. Lipids were extracted with chloroform : methanol (2 : 1). Dried material was resuspended in 300 *μ*L of chloroform : methanol (2 : 1), and analyzed by thin layer chromatography. Thin layer chromatography plates precoated with Silica Gel 60 having a thickness of 0.25 mm (MCB Manufacturing Chemists, Inc., Cincinnati, OH) were used, and the solvent system was chloroform : methanol : water (65 : 30 : 4). After lipids were separated by thin layer chromatography, radioactivity in the fatty acid and phospholipid bands was measured using a Beckman LS 7500 scintillation counter (Fullerton, CA). Phospholipase activity was expressed as percentage of total radioactivity converted into lysolecithin and fatty acids.

## 3. Results and Discussion

### 3.1. Genes that Regulate Influx of Cholesterol

According to the model proposed by Hui et al. [16], the first step in transporting cholesterol across the brush border membrane of enterocytes is uptake of cholesterol at the membrane which is mediated by the cluster determinant 36 (CD36) and scavenger receptor class B type I (SR-BI) proteins. The second step is intracellular transport of cholesterol from the plasma membrane to the endoplasmic reticulum which is mediated by the Niemann-Pick C1-like 1 (NPC1L1) protein.

There was no difference in *CD36* and *SR-BI* mRNA expression on the basal diet. Slightly lower levels of *CD36* mRNA (1.4-fold, *P* = .042) and *SR-BI* mRNA (1.5-fold, *P* = .034) were expressed in high responders relative to those in low responders on the HCLF diet (Table 2). *NPC1L1* mRNA levels did not differ between high and low responders on the basal or HCLF diet (Table 2). Therefore, the mRNA expression of proteins that mediate influx of cholesterol from the intestinal lumen into enterocytes did not show any difference between high and low responders that can explain higher cholesterol absorption in high responders on the HCLF diet.

### 3.2. Genes that Regulate Efflux of Cholesterol

The ATP-binding cassette (ABC) proteins, ABCG5 and ABCG8, are present in the apical membrane of enterocytes where they efflux cholesterol back to the intestinal lumen for excretion [17]. *ABCG5* and *ABCG8* mRNA levels were similar in high and low responders on the basal diet (Table 2). There was also no significant difference in *ABCG5* and *ABCG8* mRNA expression between high and low responders on the HCLF diet, suggesting that efflux of cholesterol back to the lumen is not impaired in high responders. Furthermore, the two ABC transporter genes were up-regulated (2-fold) in high and low responders (Table 2) consuming the HCLF diet which could limit the absorption of cholesterol and help maintain cholesterol homeostasis.

The ABCA1 protein is expressed in the basolateral membrane of enterocytes, where it effluxes cholesterol from enterocytes for the production of high density lipoprotein [6, 7]. The role of ABCA1 in transporting cholesterol to the intestinal lumen is still unclear because of conflicting data from different studies [7], and its location on the basolateral surface of enterocytes is different from ABCG5 and ABCG8. However, the *ABCA1* gene was regulated coordinately with the *ABCG5* and *ABCG8 *genes in high and low responders on the two diets, because transcription of the *ABCA1*, *ABCG5*, and *ABCG8* genes are all under the control of the liver X receptor [18, 19].

### 3.3. Cholesterol Synthesis Genes

Cholesterol synthesis is regulated by negative feedback inhibition [20]. The mRNA expression of key cholesterol synthesis enzymes was determined in the intestines of high and low responders. There were no significant changes in mRNA levels of 3-hydroxy-3-methylglutaryl-coenzyme A reductase (HMGCR), the major rate-limiting enzyme of cholesterol synthesis, after dietary challenge in high and low responders. A decrease (2-fold) in mRNA levels of 3-hydroxy-3-methylglutaryl-coenzyme A synthase (HMGCS1) was observed after the dietary challenge only in high responders. Squalene epoxidase, SQLE, is the secondary rate-limiting enzyme in cholesterol synthesis [21]. High and low responders exhibited a decrease in *SQLE* mRNA levels, 6.7-fold and 2.8-fold, respectively, after challenge with the HCLF diet. These results indicate that consumption of the HCLF diet causes an increase in cellular cholesterol in the enterocytes. Decreased expression of the cholesterol synthesis genes acts in concert with increased expression of the *ABCG5* and *ABCG8* genes to regulate cellular levels of cholesterol in the intestines of high and low responders.

### 3.4. Cholesteryl Ester Synthesis Genes

Cholesterol taken up at the cell membrane is transported to the endoplasmic reticulum where free cholesterol is converted to cholesteryl esters by the acyl-coenzyme A: cholesterol acyltransferase 2 (ACAT2) enzyme [5]. Incorporation of cholesteryl esters, triglycerides, and nascent apoB lipoproteins into chylomicrons for secretion into lymph is facilitated by the microsomal triglyceride transfer protein (MTP), such that MTP alleviates feedback inhibition of ACAT activity by cholesteryl esters [22]. In high and low responders, dietary challenge appeared to have little effect on *ACAT2* and *MTP* mRNA levels (Table 2).

### 3.5. Genes from Subtraction Cloning

Two subtracted cDNA libraries were generated by suppression subtractive hybridization together with mirror orientation selection. After differential screening of 400 clones from each of the forward- and reverse-subtracted cDNA libraries, we found most of the subtracted genes are not involved in cholesterol absorption, but two phospholipase genes (*PLA2* and *PLB*) and the gene for the intestinal bile acid binding protein (*IBABP*) could be related to cholesterol absorption, so their expression in high and low responders was further analyzed by qRT-PCR.

The *PLA2* gene encodes phospholipase A_2_ which catalyzes the hydrolysis of the ester bond at the *sn-2* position of glycerophospholipids to produce free fatty acids and lysophospholipids [23]. The *PLB* gene encodes phospholipase B (PLB) which displays broad (hence B) lipolytic activities, including phospholipase A_2_, lysophospholipase, and lipase activities [24, 25]. Similar mRNA levels of the two phospholipase genes were present in high and low responders on the basal diet. *PLA2* mRNA levels were slightly higher (1.5-fold, *P* = .0003) in high responders relative to those in low responders on the HCLF diet. Dietary cholesterol induced expression of the *PLB* gene in both high and low responders, but the increase was greater in high responders such that the levels in high responders were 3-fold (*P* = .014) higher than those in low responders. 

**Figure 1:**
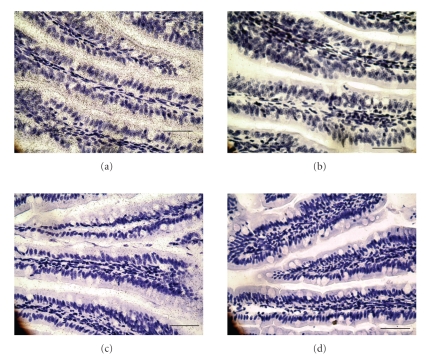
Localization of *PLB* mRNA in the jejunum of opossums fed the HCLF diet. Light-field autoradiographs of intestinal sections from the proximal third segment of a high responding opossum ((a) and (b)) and a low responding opossum ((c) and (d)). With the *PLB* antisense probe, strong hybridization signals were present in the villi of the high responder (a), but weak signals were present in the low responder (c). With the *PLB* sense probe, hybridization signals were barely detectable ((b) and (d)). Bar is 50 *μ*m.

Since total RNA was extracted from a segment of the small intestine, we studied localization of *PLB* mRNA in the proximal jejunum of opossums fed the HCLF diet by in situ hybridization. The hybridization signal was very strong with the antisense probe in a high responder (Figure 1(a)), but was much weaker in a low responder (Figure 1(c)). In contrast, only background signal was observed with the sense riboprobe (Figures 1(b) and 1(d)), showing that the antisense probe was specific for *PLB* mRNA. Opossum *PLB* mRNA was detected in the villi, which is consistent with studies in rabbits [24] and rats [25] showing by immunostaining that PLB is expressed in the brush border membrane of enterocytes.

We also measured phospholipase activity in the small intestines of high and low responding opossums. Phospholipase activity in high responders was significantly higher than that in low responders (55.9 ± 9.1% in high responders versus 20.4 ± 4.2% in low responders, *P* = .007), which is consistent with higher mRNA expression of the phospholipase genes in high responders.

Dietary cholesterol has to be incorporated into bile salt mixed micelles in the intestinal lumen so that ingested cholesterol can be transported to the brush border membrane and taken up by enterocytes [5, 6]. It has been shown that hydrolysis of phosphatidylcholine by phospholipase A_2_ and carboxyl ester lipase releases cholesterol from the bile salt micelles and facilitates the uptake of cholesterol by intestinal cells [26–28]. The *PLA2* and *PLB* genes encode enzymes that are capable of hydrolyzing phosphatidylcholine. Higher *PLA2* and *PLB* mRNA levels, as well as higher phospholipase activity, were associated with higher cholesterol absorption in high responders. It is possible that intestinal phospholipase A_2_ and phospholipase B may supplement pancreatic phospholipase A_2_ and carboxyl ester lipase in the digestion of phospholipids, and therefore higher levels of *PLA2* and *PLB* mRNA may increase cholesterol absorption in high responders.

The intestinal bile acid binding protein (IBABP) is a cytosolic bile acid transporter that shuttles bile acids absorbed at the apical membrane to the basolateral membrane of ileal enterocytes during the enterohepatic circulation of bile acids [29]. The *IBABP* gene was expressed at extremely low levels in high and low responders while they were fed the basal diet, suggesting that there is minimal uptake of bile acids in the jejunum. Feeding opossums the HCLF diet markedly induced *IBABP* mRNA levels in both high and low responders, possibly facilitating absorption of bile acids. Average *IBABP* mRNA levels were higher in high responders than in low responders, but the difference was not statistically significant due to wide variation in mRNA levels within the two groups of opossums (Table 2). Increased bile acid absorption in the jejunum could be a response to decrease cholesterol absorption on the HCLF diet since bile salt micelles are needed to solubilize cholesterol for absorption [6, 30].

## 4. Conclusions

Expression of most of the genes examined in this study was regulated similarly in high and low responding opossums to limit the absorption of excess cholesterol when they were challenged with the HCLF diet. Higher *PLA2* and *PLB* mRNA expression in high responders compared to that in low responders may contribute to higher cholesterol absorption in high responders on the HCLF diet. However, the difference in cholesterol absorption between high and low responders could be attributable to differences in hepatic and biliary cholesterol metabolism. We reported that biliary cholesterol concentrations in high responders were significantly lower than those in low responders while they were on the HCLF diet [4]. An inverse relationship between biliary cholesterol concentration and dietary cholesterol absorption was demonstrated in mice fed diets with increasing amounts of cholesterol [31]. Cholesterol absorption in the intestine appears to be a saturable process, and high concentrations of biliary cholesterol may diminish bile salt mixed micelles to solubilize dietary cholesterol for absorption [31]. Future studies will address whether a lower concentration of biliary cholesterol in high responders would allow micellar solubilization of a larger mass of dietary cholesterol, and thus absorption of a higher percentage of dietary cholesterol. 

Low concentrations of biliary cholesterol in high responders indicate that biliary cholesterol secretion is impaired. This could be due to phosphatidylcholine-poor bile which cannot solubilize a greater amount of cholesterol when high responders are fed the HCLF diet, since phospholipids are essential for secretion of cholesterol into bile [32, 33]. Up-regulation of phospholipase activities could be a response to increase the amount of phospholipids in the bile. An increase in intestinal phospholipase activities will increase the production of lysophosphatidylcholine, and an increase in lysophosphatidylcholine absorption in the intestine will in turn increase phosphatidylcholine synthesis in the liver for secretion into bile. Future studies will determine if high and low responders differ in biliary phospholipid concentrations.

## Figures and Tables

**Table 1:** Primer sequences and accession numbers.

Gene	Forward primer (5′ → 3′)	Reverse primer (5′ → 3′)	Accession number
*CD36*	CGTACAGGGTCCGCTACCTA	AGCTGCAACAGCAAGATTCA	XM_001364338
*SR-BI*	TGCTGGTCGAATCTGTCACT	CCAGGGATGGACTTCATGT	XM_001379262
*NPC1L1*	GCTTATGATGGTGCCGTGAA	CCGAAGGTCAGCTGTGATGT	EU886296
*ABCG5*	CAGCAGCGTGTTGTATTGGA	AGCCGCGCACAGCAATACC	EF599647
*ABCG8*	ACTTGACCGTCTGGGAGACTT	ACACTCCCCGCAGGTACTC	EF599648
*ABCA1*	GAGGGTGGAGGGTTGAAGA	CGAGAAAGAGGACTAGATTCCAAA	EF640974
*HMGCR*	CCATGTCAGGTGTTCGACAA	TTGCCATATTGGACGACCTT	EF599116
*HMGCS1*	GTGGGACACACATGCAACA	CATTTCCCTCTTTCTGCCATTT	EF599117
*SQLE*	TGGCAGAACCCAATACAAAGT	AAAGCCCATCTGCAACAACT	EF599115
*ACAT2*	CTTCCCGCTGTGTCCTAGTCTT	GTGTGGGGCAAAAGAGGAAGTA	EF640976
*MTP*	GAGCATCCACATATAGCCTTGA	GGTTCTCCTCACCCTCATCA	XM_001369575
*PLA2*	CTGCGGGTCTGGAACATT	GGCTCTCAGCATTGAGCATT	EC091492
*PLB*	CAATACCGAGGTCTCTCTTGGA	GTCTCTGATGTGGCTCCTGCTA	DQ875604
*IBABP*	CGTAGGTCACACCTCCGCTAGT	GGCAAGGAGTGTGAAATGGA	EC091480
*GAPDH*	GGAGAAAGCTGCCAAATACG	GAAGAGTGGGTGTCGCTGTT	EF599650

**Table 2:** Expression of intestinal genes in high and low responders fed the basal or HCLF diet.

	Basal diet		HCLF diet	
Gene	High responders	Low responders	High responders	Low responders
*CD36* ^§^	0.83 ± 0.12	0.80 ± 0.12	0.79 ± 0.12*	1.11 ± 0.31
*SR-BI*	0.81 ± 0.22	0.99 ± 0.35	0.77 ± 0.21*	1.19 ± 0.35
*NPC1L1*	1.18 ± 0.20	1.15 ± 0.15	1.08 ± 0.15	0.97 ± 0.04
*ABCG5*	0.55 ± 0.12^†^	0.48 ± 0.08^†^	1.20 ± 0.23	1.18 ± 0.27
*ABCG8*	0.55 ± 0.18^†^	0.41 ± 0.10^†^	1.21 ± 0.15	1.02 ± 0.26
*ABCA1*	0.48 ± 0.18^†^	0.47 ± 0.24^†^	1.34 ± 0.32	0.93 ± 0.25
*HMGCR*	1.33 ± 0.67	1.25 ± 0.25	0.88 ± 0.17	1.07 ± 0.21
*HMGCS1*	0.75 ± 0.26^††^	1.09 ± 0.44	0.38 ± 0.07*	0.80 ± 0.16
*SQLE*	1.01 ± 0.31^††^	1.27 ± 0.28^††^	0.15 ± 0.06*	0.46 ± 0.19
*ACAT2*	0.94 ± 0.18	0.71 ± 0.12	0.85 ± 0.10	1.05 ± 0.10
*MTP*	1.01 ± 0.10	1.04 ± 0.17	0.97 ± 0.13	1.18 ± 0.08
*PLA2*	1.22 ± 0.41	1.08 ± 0.53	1.31 ± 0.16**	0.88 ± 0.04
*PLB*	0.52 ± 0.49^†^	0.39 ± 0.15^†^	2.61 ± 1.23**	0.86 ± 0.21
*IBABP*	0.01 ± 0.01	0.02 ± 0.02	0.96 ± 1.29	0.11 ± 0.13
*GAPDH* ^§§^	672 ± 90	740 ± 85	795 ± 152	686 ± 139

^§^mRNA expression was normalized to *GAPDH* mRNA expression.

^§§^Values of *GAPDH* mRNA expression.

*Significant lower expression (*P* < .05) in high responders versus low responders on HCLF diet.

**Significant higher expression (*P* < .05) in high responders versus low responders on HCLF diet.

^†^Significant lower expression (*P* < .05) on basal diet versus HCLF diet.

^††^Significant higher expression (*P* < .05) on basal diet versus HCLF diet.
